# Feasibility of an early progressive resistance exercise program for acute Achilles tendon rupture

**DOI:** 10.1186/s40814-024-01494-4

**Published:** 2024-04-22

**Authors:** Marianne Christensen, Karin Grävare Silbernagel, Jennifer A. Zellers, Inge Lunding Kjær, Michael Skovdal Rathleff

**Affiliations:** 1 Physiotherapy and Occupational Therapy, Aalborg University Hospital, Hobrovej 18-22, Aalborg, 9000 Denmark; 2 Department of Orthopaedic Surgery, Aalborg University Hospital, Aalborg, Denmark; 3 Department of Clinical Medicine, Aalborg University, Aalborg, Denmark; 4 Department of Physical Therapy, University of Delaware, Newark, DE USA; 5 Program in Physical Therapy and Department of Orthopaedic Surgery, Washington University School of Medicine in St. Louis, St. Louis, MO USA; 6 Department of Health Science and Technology, Aalborg University, Aalborg, Denmark

**Keywords:** Achilles tendon rupture, Early functional rehabilitation, Strength resistance exercise, Feasibility

## Abstract

**Background:**

Long-term strength deficits are common after Achilles tendon ruptures. Early use of progressive resistance exercises may help reduce strength deficits, but the feasibility of this approach is unknown. The aim was to investigate the feasibility of early progressive resistance exercises regarding patient acceptability and compliance with the intervention.

**Methods:**

We recruited patients with an acute Achilles tendon rupture treated non-surgically. During 9 weeks of immobilisation with a walking boot, participants attended weekly supervised physiotherapy sessions of progressive resistance exercises and performed home exercises, consisting of isometric ankle plantarflexion, seated heel-rise, and elastic band exercises. Acceptability was evaluated using a 7-point Likert scale (1 = very unacceptable and 7 = very acceptable) with feasibility threshold at 80% of the participants rating ≥ 4. Adherence to the exercises was defined as 80% of the participants performing at least 50% of the home exercises. During the intervention, tendon healing and adverse events were monitored.

**Results:**

Sixteen participants (mean age 46 (range 28–61), male/female = 13/3) completed the intervention. Pre-injury Achilles tendon total rupture score was 98 (SD 8). All participants rated the acceptability of the exercises ≥ 5 (moderate acceptable to very acceptable) at 9- and 13-week follow-up and 9/16 rated 7 points (very acceptable). Participants performed 74% (range 4–117) of the total prescribed home exercises and 15/16 performed > 50%. One participant was not compliant with the home exercises due to feeling uncomfortable performing these independently. There were no re-ruptures, but one case of deep venous thrombosis.

**Conclusions:**

The early progressive resistance exercise program for treatment of non-surgically treated Achilles tendon rupture was feasible. Future studies should investigate the efficacy of the progressive intervention.

**Trial registration:**

The study was registered at Clinical Trials (NCT04121377) on 29 September 2019. ClinicalTrials: NCT04121377.

**Supplementary Information:**

The online version contains supplementary material available at 10.1186/s40814-024-01494-4.

## Key messages regarding feasibility

- Early progressive resistance exercises after Achilles Tendon rupture may be important to reduce long term strength deficits. However, the feasibility of this treatment strategy is unknown. This knowledge is important before engaging in a large randomised controlled trial.
- The patients found the early progressive resistance exercises highly acceptable, and compliance with the exercises was high.
- Given the feasibility of the early progressive resistance exercises, it is recommended to investigate the efficacy of the exercises.

## Background

Patients with an acute Achilles tendon rupture are at risk of long-term deficits in muscle strength and function of the lower leg. These deficits remain up to 10 years after the initial rupture [1–3]. A lack of lower leg function (e.g. plantar push off) can negatively impact both work and sports participation [4–8]. Not being able to carry out normal daily activities such as running or performing normal jobs as before the injury may impact both quality of life and physical activity levels [9]. Reducing the negative long-term consequences are thus of utmost importance.

The current treatment approach enforces the use of early functional rehabilitation (EFR) in treatment for Achilles tendon rupture during the first 8 weeks of treatment. This approach is recommended, irrespective of the primary treatment (e.g. surgical or non-surgical) [10–14]. EFR aims to stimulate tissue and motor function to minimise the loss of muscle strength that normally occurs during immobilisation. Laboratory studies in animals demonstrate that early loading improves tendon healing, and clinical studies also point towards similar benefits in humans [15–17].

We recently conducted a systematic review investigating the EFR protocols used in the first 8 weeks of treatment. We discovered very heterogeneus intervention protocols and a lack of details on exercises and progression criteria [18, 19]. The individual components of EFR ranged from early weight-bearing and controlled ankle/foot range of motion exercises to specific resistance exercises. The timing of when to initiate exercises, weight-bearing recommendations, and range of motion restrictions varied between studies with no clear protocols. An important finding was that studies mainly focused on mobilisation of the ankle and did not include specific resistance exercises that would load the Achilles tendon.

Based on the lack of specific resistance exercises, we developed a specific protocol together with expert clinicians and based it on inputs from patients and expert clinicians. The aim of the protocol was to initiate early progressive resistance exercises as part of EFR to combat loss of muscle strength and function during the 9 weeks with a walking boot. As this protocol differs substantially from most previous studies [19] in particular for non-surgical treatment, it needs to be tested for feasibility and acceptability before it can be tested in larger trials. The aim of this study was to test the feasibility of an early progressive resistance exercise program for patients with Achilles tendon rupture treated non-surgically. Feasibility in this study was defined as successful patient acceptability and compliance of the exercise intervention.

## Methods

### Study design

This study was designed as a single group, interventional feasibility study. The reporting of the study follows the CONSORT 2010 statement, extension to randomised pilot and feasibility trials [20] (Additional file 1). Results will be used in a subsequent randomised controlled trial (RCT), but since the main focus is the patient’s willingness and adherence to the exercise program, there were no comparator used in this feasibility study. The same experienced physiotherapist (primary investigator) performed the intervention, monitored the tendon healing process, and collected follow-up data. No blinding was applied for the follow-up.

The study complied with the principles of Helsinki Declaration. It was approved by the Regional Committee on Health Research Ethics in North Denmark Region (N-20180072) and the protocol was prospectively registered in Clinical Trials (NCT04121377).

### Participants

Inclusion criteria were patients with acute total Achilles tendon rupture treated non-surgically, aged between 18 and 65 years, able to speak and understand Danish, and able and willing to participate in the intervention. We excluded patients with delayed diagnosis and treatment > 3 days from injury, due to risk of tendon elongation [21, 22]. This is a standard criterion for RCTs comparing treatments for Achilles tendon rupture [23–25]. We further excluded patients with insertional Achilles tendon rupture on calcaneus, high rupture in the musculo-tendinous junction of the triceps surae, previous Achilles tendon rupture or other conditions in either leg causing lower leg disability (pain, deficits in strength or range of movement), treatment with fluroquinolones or corticosteroids within the last 6 months [24, 25], diabetes, and severe medical illness (American Society of Anesthesiologists (ASA) score higher than or equal to 3) [26].

Participants were recruited from the Orthopaedic Outpatient Clinic at Aalborg University Hospital Denmark from October 2019 to January 2020. The primary investigator received referrals from the Emergency Department and contacted potentially eligible participants to schedule an information meeting within a week from the start of treatment. A trial informational document was emailed at least 24 h before the meeting, where the informed consent was obtained.

### Intervention

#### Patient and clinician involvement

We developed the intervention using the findings from a systematic review [18, 19], alongside suggestions from informal focus group meetings. To ensure feasibility of the exercise program, we invited patients to discuss facilitators and barriers for performing exercises in the early rehabilitation from their own perspective. The patients added that it would be acceptable to perform the progressive exercises if it was supervised by a physiotherapist. Physiotherapists, orthopaedic surgeons, and a rheumatologist, all with experience in treating patients with Achilles tendon ruptures, were invited to discuss the literature, clinical relevance, and rationale for an early progressive resistance exercise program. The clinicians emphasised that it was important to monitor the tendon healing due to the risk of re-rupture or tendon elongation.

#### Development of the intervention

The intervention description followed the Template for Intervention Description and Replication (TIDieR) [27] and The Consensus on Exercise Reporting Template (CERT) [28]. The strength exercises were supplied as an add-on to the standard program which contained more general exercises with controlled range of motion and exercises for the whole leg (Table 1) (Extended version in Additional file 2).

**Table 1.**
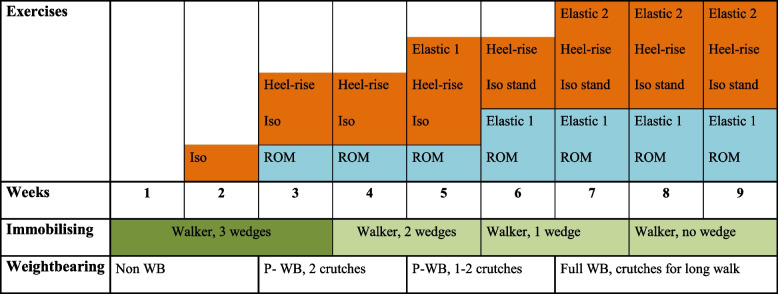
Schedule of exercises and immobilisation

Blue, standard exercise program; orange,  add-on exercises; *Iso* isometric contractions, *ROM* controlled range of motion; *Heel-rise*, seated heel-rise; *Elastic 1*, light load (yellow-red); *Elastic 2*, progression of load to stronger elastic band (red, blue, green); *WB* weight bearing, *P-WB* partial weight bearing

**Fig. 1:**
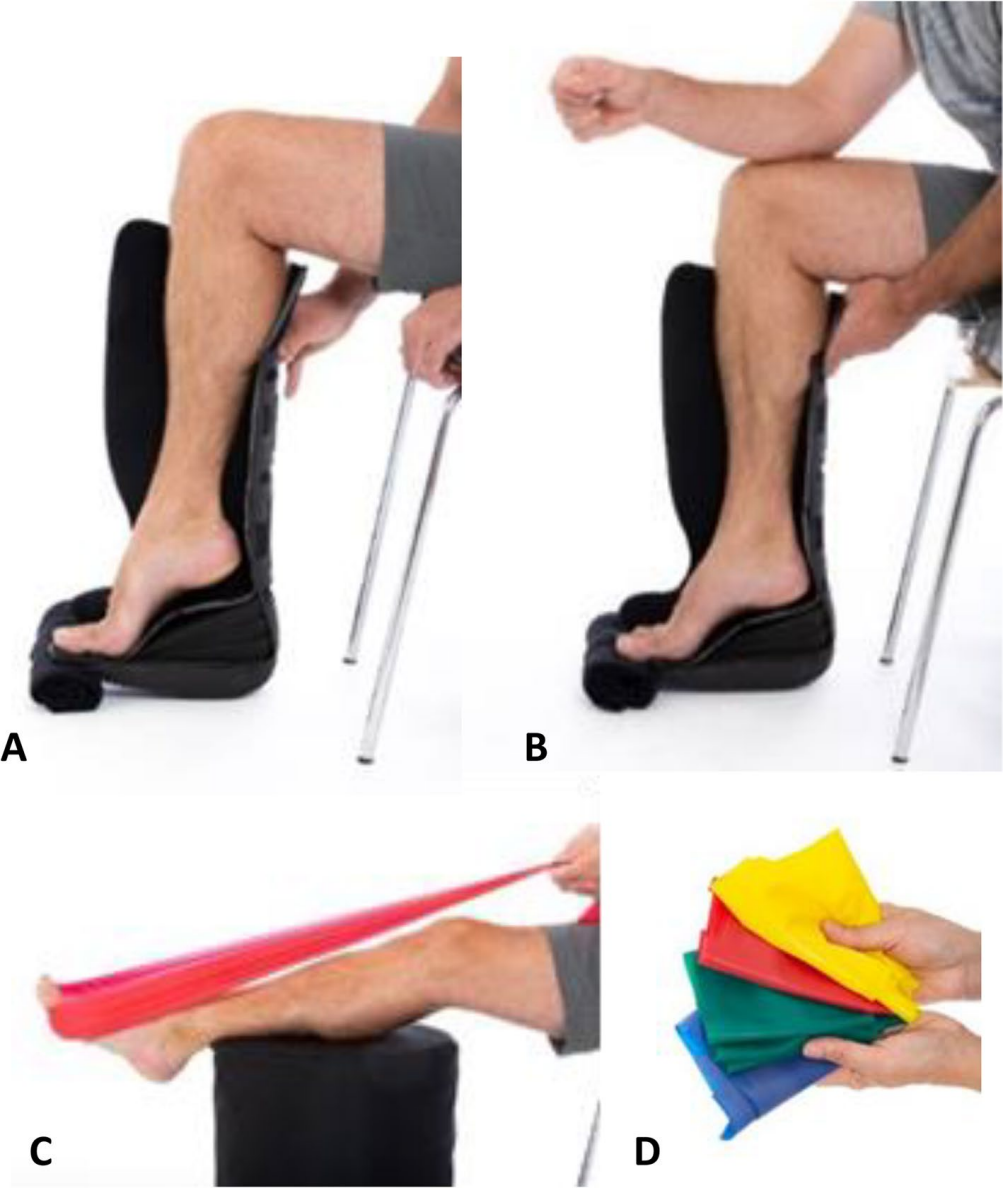
Illustration of exercises. **A** Seated heel-rise in orthosis. **B** Seated heel-rise in orthosis with extra load from upper torso. **C** Plantar flexion with elastic band. **D** Elastic bands with different level

The new program consisted of three exercises (Fig. 1) with earlier starting time and gradually more progression to exercises with higher loads than the standard program: (a) isometric ankle plantar flexion in a closed walking boot performed every hour, (b) seated heel-rise performed five times per day in open walking boot with wedges according to week number, and (c) resistance exercises with elastic band five times per day. Exercise program and dosage of the exercises are described in additional files (Additional file 3, Additional file 4).

One week after diagnosis, the intervention started with a daily home program and weekly sessions (once per week) with a physiotherapist to assess the acceptability of the program and to monitor the healing of the Achilles tendon. To protect the tendon while doing range of motion of the foot, dorsiflexion was restricted beyond neutral (0° of dorsiflexion). Progression was performed individually and with a gradual progression [29]. The Borg CR10 scale of perceived exertion was used to guide the participant to progress or regress the load in each exercise [30], with the recommended level being “easy” to “hard” (2–5/10). It was emphasised that the exercises should not cause sudden or severe pain in the tendon, but muscle soreness could be expected. Ultrasound examination of the leg muscle contractions was planned to confirm facilitation of the triceps surae muscles.

The intervention took place at the Physiotherapy and Occupational Therapy Department at Aalborg University Hospital. One experienced physiotherapist supervised in all sessions.

#### Outcomes

The focus of this study was to test the feasibility of the exercise intervention by setting values for fulfilment of a range of outcomes and process variables deemed of importance to patients and safety. There were two primary outcomes of interest, namely patient acceptability (willingness) and adherence to the intervention. Follow-up measurements occurred at the end of weeks 9 and 13. Acceptability was also measured after each weekly exercise session and the participants registered all exercises for each week in a training journal.

The exercise program was considered feasible if:

- (A) The acceptability of the exercise program was 80%. Defined as: ≥ 13/16 participants rating the acceptability of the intervention as “acceptable” on a 7-point Likert scale [31].
- (B) The adherence to the exercise program was 80% of participants performing at least half of the home exercises. Defined as: ≥ 13/16 participants performing ≥ 50% of the home exercises until the end of week 9.

##### Rationale for (A) Acceptability

Patient acceptability was not a measure of whether symptoms had improved to normal physical function or any other satisfactory level at the specific time. Rather, acceptability was defined by how well the intervention matched their expectations of an exercise program in this early phase and how they tolerated performing the exercises. The intervention program was categorised as “Unacceptable” if rated as the three lower scores (very unacceptable, unacceptable, slightly unacceptable) and categorised as “Acceptable” if rated as the four higher scores (“neither acceptable nor unacceptable” to “very acceptable”).

##### Rationale for (B) Adherence

The participants registered the number of each of the three resistance home exercises they performed each day in a training journal. Compliance was defined as the total number of home exercises performed in percent of the prescribed numbers for each participant. The timeframe was from the day they started the exercises to the end of week 9.

##### Clinical relevance

The exercise program was based on home exercises that should be easy to understand and perform without the need for physiotherapist supervision, as this will be both time-consuming and incur higher costs. Total (100%) compliance to the exercise program cannot be expected. Prior studies have found exercise compliance to be very poor, with values as low as 45% or less for chronic diseases [32–34]. The magnitude of this exercise program leaves room for individuals with higher physical level or motivation, but the success rate reflects the reality of compliance for most individuals.

#### Secondary outcomes

##### Self-reported outcomes

The Achilles Tendon total Rupture Score (ATRS) is the most common used self-reported outcome measure and it contains 10 questions about physical performance in a scale from 0 to 10 with a maximum total score of 100 [35]. International Physical Activity Questionnaire (IPAQ) short form Danish version which consists of seven items concerning physical activity during the past week estimating the total weekly physical activity measured in MET-minutes per week (metabolic equivalent of task) [36].

The Tampa scale of Kinesiophobia (TSK) 17-item version is used as a measure for fear of movement. As it was developed for chronic pain, we subsequently asked the participants to rate the appropriateness of the score on a Likert scale [37].

##### Objective outcomes

The Achilles tendon resting angle (ATRA) was measured with a standard goniometer [38]. Ultrasound imaging was used to measure Achilles tendon length following the Copenhagen Achilles Length Measurement (CALM) [39] and Achilles tendon cross-sectional area measured at the midpoint of the rupture site [40]. Muscle endurance was measured in the seated position with the MuscleLab measurement system (Ergotest Technology, Oslo, Norway) with an external weight equal to 50% of bodyweight [41]. Ability to perform a single standing heel-rise on the injured leg with a minimum height of 2 cm [37].

#### Follow-up visits

Follow-up measurements were scheduled at the end of weeks 9 and 13. At the 9-week visit in the outpatient clinic treatment recommendations for walking changed and the participants transitioned from wearing a walking boot to protective weightbearing in shoes with 1 cm heel inserts for 3 months. In the first 2 weeks, it was advised to use crutches. ATRS and IPAQ were not measured at the 9-week follow-up due to the physical activity restrictions. Muscle endurance was not measured at the 9-week follow-up for safety reasons to protect the tendon from overuse at the time of transition.

#### Adverse events

Serious and minor adverse events were registered in a pre-defined list based on Common Terminology Criteria for Adverse Events [42] and the participants were asked open-ended questions at the exercise sessions. Each exercise session began with assessing symptoms, adverse events, and the progression of the tendon healing. Participants also registered adverse events in questionnaires at 9- and 13-week follow-up. Serious adverse events were re-rupture, non-union of the tendon, or deep vein thrombosis (DVT). Muscle soreness or mild pain was considered inevitable and normal when initiating exercises after a period of immobilisation.

At the scheduled 2 weeks visit to the outpatient clinic and at the weekly exercise sessions, all participants were clinically assessed as part of the routine standard of care procedures to assess response to treatment and risk for adverse events (no palpable gap at the rupture site, the foot positioned in equinus, some tenderness at the tendon allowed but no pain, some swelling of the surrounding tissue allowed but no large edema).

A safety committee assessed and graded symptoms that were not within normal injury or exercise reactions. Adverse events was graded 1 to 5 according to the Common Terminology Criteria for Adverse Events v4.03 [42]. In case of serious adverse events, it would be decided whether to delay the proceeding of the intervention or withdraw the participant from the study.

### Sample size considerations and statistical methods

Since this was a feasibility study and we primarily were interested in estimates of feasibility and acceptability, no formal sample size calculation was performed [43]. Estimated number of participants were 16, which seemed appropriate to evaluate the feasibility outcomes [44]. The overall incidence of Achilles tendon ruptures presenting at our hospital are approximately 80 per year.

Acceptability is presented as median and quartiles with criteria for fulfilment defined as 80% of participants scoring “acceptable”. Adherence was calculated for each participant by combining the number of completed home exercises for the three resistance exercises in percentage of the total prescribed number for the participant. The calculations were based on “number of exercise interventions per day” in Toigo and Boutellier item 5, which consists of prescribed sets (item 3) and repetitions (item 2) for each exercise (see exercise descriptors in Additional file 4). The number of prescribed exercises varied between participants, e.g. availability of appointments as no appointments in weekends or participants being unavailable. The criteria for fulfilment was 80% of participants performing ≥ 50% of the exercises. Descriptive information was registered at baseline: age, sex, height, weight, IPAQ, and ATRS. Baseline and follow-up data is presented as means with standard deviations. Reasons for exclusion or withdrawal are summarised. Safety is presented as number and percentage of participants reporting adverse events divided in major and minor events. Time from treatment start to beginning exercises is reported as mean days and range. The intention-to-treat principle was used for analyses and all participants were included in the analyses regardless of the acceptability or compliance to the intervention. Data were entered in REDCap hosted at Aalborg University Hospital [45]. If the feasibility definition for acceptability and adherence are not met, we will not pursue a definitive randomised trial.

## Results

**Fig. 2:**
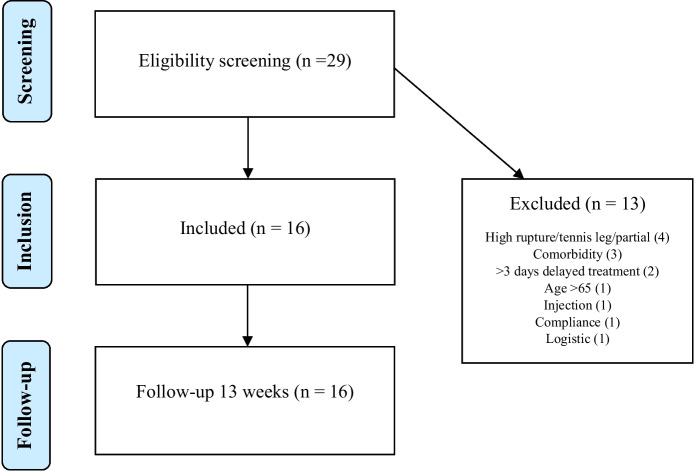
Study inclusion flow diagram

From October 9, 2019, to January 29, 2020, 29 patients were assessed for eligibility and 16 were included (Fig. 2) and all participants completed 9- and 13-week follow-up. The mean(SD) age was 46(12.0) years and the male/female ratio was 13/3 (Table 2).

**Table 2:** Demographics

Characteristics	Baseline *n* = 16
Age	46 (12)
Left/right	7/9
Sex male, *n*(%)	13 (81)
Height, cm	181 (7)
Weight, kg	83 (12)
BMI	25 (3)
ATRS (pre-injury score)	98 (8)
IPAQ (pre-injury score)	
MET min/week	3246 (736;4207.5)
Categorized (low/moderate/high)	5/3/8
TSK (pre-injury score)	41 (7)
Injury type	
By sports, *n*(%)	12 (75)
Recently resumed sports, *n*(%)	5 (31)

Data are presented as numbers (percentage), mean(SD) or median(interquartile range)

*BMI* body mass index, *ATRS* Achilles Tendon total Rupture Score, *IPAQ* International Physical Activity Questionnaire, *MET* metabolic equivalent of task, *TSK* Tampa Scale of Kinesiophobia

### Feasibility results

All participants found the exercise program moderate to very acceptable at the 9- and 13-week follow-up (Table 3). During the sessions, ratings of “moderate unacceptable” were registered by one participant at the first exercise session and by another participant at the fifth session; however, the overall acceptability fulfilled the feasibility threshold of 80% of the participants rating ≥ 4 on the Likert scale.

**Table 3:** Rating of acceptability of the exercises on a 7-point Likert scale (number of participants)

Acceptability	9 weeks	13 weeks
7 Very acceptable	10	9
6 Acceptable	5	6
5 Moderate acceptable	1	1
4 Neither nor		
3 Moderate unacceptable		
2 Unacceptable		
1 Very unacceptable		

Participants completed a mean(SD) of 486 (171) (range: 24–825) number of home exercises during the 9-week intervention period. The participants performed 74% (range 4–117) of the total prescribed number of home exercise and all but one performed ≥ 50%. Most often it was the isometric exercises that were not completed as many times as prescribed (every hour when awake = 12 times/day in 4 weeks). One participant was not compliant with the home exercises due to feeling uncomfortable performing these independently. Overall adherence to the exercises fulfilled the criteria of 80% performing at least 50% of home exercises.

### Other outcomes

Secondary outcomes for patient reported outcomes and physical measurements are presented in Additional file 5.

### Complications and changes during study

During the study period, there was one serious adverse event with a DVT occurring 7–10 days after injury. There were no re-ruptures or non-unions. Three participants had accidents with the crutches during the first weeks, the falls resulted in momentary pain but no trauma or re-ruptures. Three reported short-lived pain for a few hours when walking after the second wedge was removed (after 5 weeks). In 10 cases, the participant received extra advice on preventing edema due to swelling of the lower leg/ankle.

The proposed ultrasound examination of muscle contractions in the seated heel-rise exercise was found to be impractical as it was too difficult to maintain the foot position in equinus. A simple palpation of the muscles while performing the exercises worked well and aided participant confidence. Equipment malfunction/repair resulted in missing data for muscle endurance for 9 of the 16 participants.

## Discussion

We found that the new program consisting of early progressive resistance exercises embedded within EFR was highly acceptable, and compliance was high. These findings underscore the need for future trials to investigate the clinical benefits and test if this type of intervention can prevent loss of muscle strength and function and improve patient outcomes.

### Explanations of findings

Overall, the acceptability was high and fulfilled the feasibility threshold of 80% of the participants rating ≥ 4 on the Likert scale. In choosing the cutoff, it was more important that the participants did not rate the exercise program as “unacceptable” rather than aiming for very high ratings. Some uneasiness was to be expected with an acute tendon trauma where the exercises occur at the same time as the early phase healing of the tendon, but during the nine sessions, there were only two ratings that fell just below the cutoff.

Compliance with the exercise program on average was 74% of the proposed exercises. This is an acceptable amount considering the high number of daily exercises and the fact that adherence to exercise protocols is generally reported to be very low [46]. In our study, there was a wide range of adherence to the home exercises (4–117%), indicating that factors other than patient education and physiotherapy also play a role in incorporating exercises into daily routines. The exercise program was designed to include individual progression as we anticipated differences in physical activity levels and the most important goal was that all participants could find a level and self-progress to avoid a ceiling effect for those able to add on more load. The goal of isometric exercises every hour was particularly difficult to reach when the participants became more mobile and were walking. It is advisable to lower the daily recommended number of isometrics when the activity level and weight-bearing increases.

When asked, patients with Achilles tendon rupture mention the fear of re-rupture and feeling the “pop” of the tendon as the worst thing that could happen again. This could influence the willingness to participate in the early exercises. In screening for eligibility for the study, no patients declined due to fear of the exercise intervention. During the intervention, one participant performed very few home exercises, but still attended physiotherapy sessions. It could be that the reassurance that completing any number of exercises is beneficial and gave more confidence in returning to activity of daily living as an alternative. The TSK is used and validated for chronic back pain, but has previously been used for Achilles tendon rupture evaluation, where the questions about kinesiophobia were associated to patient reported outcomes and physical activity level [37].

The focus of this intervention was to increase the load on the muscle–tendon unit while at the same time protecting the healing tendon. Substantial evidence shows risk of re-rupture, tendon lengthening, and reduced physical function after Achilles tendon rupture [21, 47, 48], but we did not find any serious adverse events to the Achilles tendon. There was one DVT occurring at 7–10 days after the rupture, but this was more likely to be associated with immobilisation than the early start of the exercises which would instead enhance the circulatory effects.

### Comparison with previous findings

A scoping review revealed that the general descriptions of early exercise programs for Achilles tendon rupture were lacking important information such as type, time of application, frequency, intensity and progression of the exercises [19]. Based on the potential of early progressive resistance exercises to facilitate tissue repair and minimise loss of strength, optimising exercises is required to address the need for improvement of muscle and tendon strength and function while also ensuring that the exercises are well described to be able to replicate the intervention. This study is the first to investigate the feasibility of an early progressive resistance exercise program for Achilles tendon rupture. Former studies have investigated early functional rehabilitation in the form of controlled foot motion [11, 49, 50], but very few had emphasis on the facilitation of the muscle–tendon strength [23].

## Limitations

This study was a feasibility study and not designed to pilot the randomisation and, therefore, it was not investigating the effects of the exercise intervention. Feasibility of the physiotherapist prescription of exercises or the feasibility in a health care system perspective could be relevant but was outside the scope of this study. The design with weekly sessions gave the possibility of prospectively monitoring the participants acceptability of the exercises in real-time to minimise any re-call bias of longer follow-up. On the other hand, there was a risk of performance bias towards a higher adherence to the program due to the repeated attention from the investigator. Reporting of home exercises in a training diary is prone to be overestimated [34]. However, the pattern of reporting observed in this study seems to align with expected, realistic patterns due to the wide range of completed home exercises, from very low to exceeding the proposed maximum. Feeling safe in an environment with access to an experienced physiotherapist and thorough information on the aim of the study could prompt a truthful reporting.

## Conclusion

The present progressive resistance exercise program for early treatment of non-surgically treated Achilles tendon rupture was feasible. The participants found the exercise program highly acceptable, and compliance with the exercises was high. Future studies should investigate the efficacy of the progressive intervention.

### Supplementary Information

**Additional file 1:** CONSORT Checklist

**Additional file 2:** Schedule of the exercises

**Additional file 3:** Exercise information

**Additional file 4:** Exercise descriptors

**Additional file 5:** Secondary outcomes

## Data Availability

The datasets used during the current study are available from the corresponding author on reasonable request.

## Abbreviations

EFR: Early functional rehabilitation
CONSORT: Consolidated Standards of Reporting Trials
RCT: Randomised controlled trial
RM: Repetitive maximum
ATRS: Achilles Tendon total Rupture Score
IPAQ: International Physical Activity Questionnaire
MET: Metabolic equivalent of task
TSK: The Tampa scale of Kinesiophobia
ATRA: The Achilles tendon resting angle
CALM: Copenhagen Achilles Length Measurement
DVT: Deep vein thrombosis
