# Supplementary material for: Feasibility of an early progressive resistance exercise program for acute Achilles tendon rupture
Source: Pilot Feasibility Stud. 2024 Apr 22;10:66. doi: 10.1186/s40814-024-01494-4 (PMC11034137; doi:10.1186/s40814-024-01494-4)
Supplement: Supplementary file 2 — Additional file 2: Schedule of the exercises [file 40814_2024_1494_MOESM2_ESM.pdf]

Additional file 2. Exercise program schedule – extended version. Feasibility study.

Exercises									
							Elastic 2	Elastic 2	Elastic 2
					Elastic 1	Heel-rise	Heel-rise	Heel-rise	Heel-rise
			Heel-rise	Heel-rise	Heel-rise	Iso stand	Iso stand	Iso stand	Iso stand
			Iso	Iso	Iso	Elastic 1	Elastic 1	Elastic 1	Elastic 1
		Iso	ROM	ROM	ROM	ROM	ROM	ROM	ROM
**Weeks**	**1**	**2**	**3**	**4**	**5**	**6**	**7**	**8**	**9**
**Immobilizing**	Walker, 3 wedges			Walker, 2 wedges		Walker, 1 wedge		Walker, no wedge	
**Weight bearing**	Non-WB		P- WB, 2 crutches		P-WB, 1-2 crutches		Full WB, crutches for long walk		
**Rationale**		A.<br>Facilitation		B.<br>Initiation of load			C.<br>Progression to more load		
**Total exercise dose***		Facilitation of muscle activity by isometric contraction without ROM		Slow controlled movement to avoid peak forces on tendon while prioritizing long TUT to maintain/increase strength			Improve strength (go to failure IF THEY ARE comfortable with it)		
**Progression***		Progress from A to B:<br>If patient feels comfortable doing facilitating exercises<br><br>If clinical screening is OK at "2-week follow-up" (no tendon gap, equal ATRA, no pain during exercises).		Progress from B to C:<br>If patient feel comfortable doing "initiation of load" exercises.<br><br>If exercises are well-managed<br>a. with regards to Toigo & Boutillier descriptors**,<br>b. without persistent pain or discomfort during and after exercises,<br>c. no compensatory movements while performing exercises.			Progress from C to exercise and mobilize without walking boot:<br>The decision to discontinue the walking boot is done at the Outpatient clinic.<br><br>The exercises should provide the foundation for the patient to be confident in future decisions of choosing sufficient amount of load to improve the muscle performance while still avoiding too much strain on the healing tendon (i.e., avoid strenuous load, avoid stretching in dorsiflexion with load)		

Blue: Standard exercise program. Orange: Add-on exercises. Iso: Isometric contractions. ROM: Controlled range of motion. Heelrise: Seated heelrise. Elastic 1: Light load(yellow, red). Elastic 2: Progression of load to stronger elastic band (red, blue, green). WB: Weight bearing. P-WB: Partial weight bearing. ATRA: Achilles Tendon Resting Angle

*Exercise description in Additional file 3. **Information on Exercise descriptors in Additional file 4

Rationale for development of the intervention

The magnitude of this exercise program was designed to leave room for patients with higher physical level or motivation, but the success rate should reflect the reality of compliance for most patients. The exercises were designed to facilitate muscle activity in the early phase of the immobilization period and subsequently progress the load to strengthen the muscles within precautions for the healing tendon. To avoid compensatory activity from m. flexor hallucis longus, instructions were to extend the first toe before plantar flexing the ankle. The load of the strength exercises progressed from isometric contraction without external load to strength resistance exercises with 10-20 RM (repetitive maximum). Each exercise could progress with added weight or stronger elastic band. Progression was performed individually and with a gradual progression. From 2 to 10% is generally accepted as a reasonable progression in resistance training and for exercise therapy for painful tendinopathy and even though specific research on Achilles tendon rupture is lacking is seems a safe precaution to prescribe in the early rehabilitation of Achilles tendon rupture.
