# Supplementary material for: Feasibility of an early progressive resistance exercise program for acute Achilles tendon rupture
Source: Pilot Feasibility Stud. 2024 Apr 22;10:66. doi: 10.1186/s40814-024-01494-4 (PMC11034137; doi:10.1186/s40814-024-01494-4)
Supplement: Supplementary file 3 — Additional file 3: Exercise information [file 40814_2024_1494_MOESM3_ESM.pdf]

Additional file 3. Exercise description. Feasibility study

Exercise 1. Isometric contraction in week 2

Starting position: Seated with closed walker boot.

Instruction: Press the ball of your foot against the bottom of the walker boot. You should be able to feel a tension in the muscles in the leg – like doing a heel raise, but without doing it. Before you start you can lift the big toe to avoid using the muscles from the toes. If the foot moves too much inside the walker boot you should tighten the straps.

Dosage:

- Repetitions:
  - 5 isometric contractions of 5 seconds duration.
  - Repeat 5 times with 5 seconds between each set.
- How often: Each hour during day and evening
- Load:
  - Aim for a light load where you do 5 contractions even if feel you can do 15-20
  - On Borg scale aim for “2 easy” in the beginning and gradually increase to “5 heavy”
  - Beginning at week 4, you can reduce the frequency to 5 times daily
- Progression
  - From week 6 you can use the isometric contraction in a standing position. Move your body a little forward so you feel the weight on the forefoot and tension in the leg muscles.

Exercise 2. Seated heel-rise. Starts after the 2-week outpatient clinic visit

Basic exercises: Exercise 1 isometric as before. Controlled foot movement from the information pamphlet (you will be instructed at the visit)

Starting position: Seated on a chair with your knee bent to 90 degrees. Use a higher chair or a cushion if the knee is too bent. Use one hand to support the walker boot on the back while you open the straps on the boot. Make sure there is no gap between the leg and the boot during the exercise.

Instruction: Lift up the big toe to avoid too much activity in the toe muscles. Lift up the heel from the wedges. Press into the ball of your foot and not the toes. You should be able to feel the tension/contraction in the muscles. Lift the heel up from the wedges. Make sure that you are using the leg muscles and not pulling up the leg with your thigh muscles.

Dosage:

- Repetitions:
  - 10-15 heel-rise repetitions
  - Repeat 3 sets with 10 seconds between each set
- How often: 5 times daily
- Load:
  - Aim for a light load in the beginning where you do many repetitions (light enough to do 15-20 repetitions) and later increase the load and do less repetitions.
  - On Borg scale aim for “2 easy” in the beginning and gradually increase to “5 heavy”
- Start and progression:
  - (write the individual level)
  - Increase in load can be from upper body when leaning with elbow on the knee or with extra weights (sandbag, weights, water bottles)

Exercise 3. Resistance exercise with elastic band. Start in week 5

Basic exercises: Exercise 1 and 2 as before. Use the controlled foot movement from the information pamphlet as warm-up.

Starting position: Seated with the knee straight on a chair or on the couch with the heel hanging off the edge. Take of the walking boot. Place the elastic band around the forefoot and tighten the band with your hands, but do not pull the foot past a neutral position (90 degree angle). Roll the elastic band an extra loop around the forefoot for a firmer grip.

Instruction: Lift up the big toe and then push the forefoot down on the elastic band. Push as far as you can. Hold the position. Then slowly move the foot back to neutral position while you keep the elastic band in tension. Be careful not to pull the elastic band too hard.

Dosage:

- Repetitions:
    - 10-15 push on the elastic band
    - Aim for 3 seconds pushing down, hold the position for 2 seconds and use 3 seconds on the return to neutral.
    - Repeat 3 sets with 10 seconds rest between sets.
- How often: 5 times daily
- Load:
    - Aim for a light load in the beginning where you do many repetitions (light enough to be able to do 15-20 repetitions) and later increase the load and do less repetitions
    - On Borg scale aim for “2 easy” in the beginning and gradually increase to “5 heavy”
- Start and progression:
    - (write the individual level)
    - Increase the load by tightening the elastic band or change to a higher level elastic band

Borg scale of perceived exertion	
0	No Exertion
1	Very light
2	Slight
3	Moderate
4	Somewhat severe
5	Severe
6	
7	Very severe
8	
9	Very very Severe
10	Maximal
