# Supplementary material for: Feasibility of an early progressive resistance exercise program for acute Achilles tendon rupture
Source: Pilot Feasibility Stud. 2024 Apr 22;10:66. doi: 10.1186/s40814-024-01494-4 (PMC11034137; doi:10.1186/s40814-024-01494-4)
Supplement: Supplementary file 4 — Additional file 4: Exercise descriptors [file 40814_2024_1494_MOESM4_ESM.pdf]

Additional file 4. Table of the three additional exercises in the Feasibility study.

**Toigo & Boutellier** exercise descriptors	**Isometric contraction**	**Seated heel-rise**	**Elastic band**
X1 Load magnitude	15-20 RM	15-20 RM<br>Progress to 15RM week 6/7<br>First step is to increase repetitions and second step is to add more load	15-20 RM<br>Progress to 15RM week 6/7<br>First step is to increase repetitions and second step is to add more load
X2 number of repetitions	5	10-15	10-15
X3 number of sets	5	3	3
X4 rest btw sets	5 sec	10 sec	10 sec
X5 number of exercise interventions	Every hour (approx 15 hours per day) for week 2-3,<br>then change to 5 times per day in week 4-9 as warm-up exercise.	5 per day	5 per day
X6 duration of the experimental period	Week 2 to 9	Week 3 to 9	From week 5 to 9
X7 Fractional and temporal distribution of the contraction modes per repetition and duration (s) of one repetition	5 sec	3s shortening<br>2s isometric<br>3s lengthening	3s-2s-3s<br>(Focus is on the concentric phase. Important not to push beyond the neutral position in the eccentric phase)
X8 Rest in-between repetitions	2	2	2
X9 Time under tension	125 s per session<br>1875 s per day (hourly)<br><br>625 s per day (5 times)	240s per session<br>1200s per day	240s per session<br>1200 s per day
X10 Volitional muscular failure	No	No	No
X11 Range of motion	No range of motion. The foot is immobilized in equinus according to the number of wedges.	From plantarflexed foot position on the wedges to more plantarflexed, when performing the heel-rise	Dorsiflexion above neutral is not allowed. Full plantarflexion allowed.

X12 Recovery time in-between exercise sessions	1 hour	3 hours	3 hours
X13 Anatomical definition of the exercise (exercise form)	Isometric contraction is performed inside the walking boot with the foot in equinus according to the weekplan. If the foot moves too much inside the walker boot you should tighten the straps.<br><br>Press the ball of your foot against the bottom of the walker boot. You should be able to feel a tension in the muscles in the leg – like doing a heel raise, but without doing it. Before you start you can lift the big toe to avoid using the muscles from the toes.	Seated heel-rise with leg in walking boot with "the wedges of the week". Seated on a chair with your knee bent to 90 degrees. Use a higher chair or a cushion if the knee is too bent. Use one hand to support the walking boot on the back while you open the straps on the boot. Make sure there is no gap between the leg and the boot during the exercise.<br><br>Lift up the big toe to avoid too much activity in the toe muscles. Lift up the heel from the wedges. Press into the ball of your foot and not the toes. You should be able to feel the tension/contraction in the muscles.<br>Lift the heel up from the walker/wedges. Make sure that you are using the leg muscles and not pulling up the leg with your thigh muscles.<br>Progression: add weight on knee (either sandbag/rice, water bottle, upper body.	Seated with the knee straight on a chair or on the couch with the heel hanging off the edge. Take of the walking boot. Place the elastic band around the forefoot and tighten the band with your hands, but do not pull the foot past a neutral position (90 degree angle). Roll the elastic band an extra loop around the forefoot for a firmer grip.<br>Lift up the big toe and then push the forefoot down on the elastic band. Push as far as you can. Hold the position. Then slowly move the foot back to neutral position while you keep the elastic band in tension. Be careful not to pull the elastic band too hard.<br>Progression: Increase the load by tightening the elastic band or change to a higher level elastic band.

Borg scale – Perceived exertion during exercise (/10) is used to guide the patients to progress or regress the load in each exercise. The recommended level being "easy" to "hard" (2-5/10). It is emphasized that the exercises must not cause sudden or severe pain in the tendon, but muscle soreness is to be expected.

Progression:

Progression of the exercises will be a continuous process. There are three main phases: facilitating, initiation of load, progression to more load. When progressing to the next phase or new type of exercise the following criteria has to be observed:

The load magnitude of the present exercise should be accomplished with the patient feeling comfortable doing the exercises and without persistent pain or discomfort during and after exercises.
