# Supplementary material for: Feasibility of an early progressive resistance exercise program for acute Achilles tendon rupture
Source: Pilot Feasibility Stud. 2024 Apr 22;10:66. doi: 10.1186/s40814-024-01494-4 (PMC11034137; doi:10.1186/s40814-024-01494-4)
Supplement: Supplementary file 5 — Additional file 5: Secondary outcomes [file 40814_2024_1494_MOESM5_ESM.pdf]

Additional file 5. Secondary outcomes. Feasibility study

Results

At the 13-week follow-up, ATRS and IPAQ were lower than the pre-injury score reflecting the early stage after immobilisation. The TSK did not change from baseline to 9 weeks and decreased by two points at the 13-week follow-up. The relevance of the TSK questionnaire was rated in the top three categories of the 7-point Likert scale by 94% of the participants at the 9- and 13-week follow-up.

Table 4. Secondary outcomes at 9- and 13-week follow-up timepoints

Outcome	9 weeks	13 weeks	
ATRS	-	40(18)	
IPAQ<br>-MET minutes/week. Median(iqr)<br>-Category low/moderate/high	 -<br>-	 2201 (1230;6719)<br>2/7/7	
TSK<br>-TSK score<br>- Rating appropriateness: Top three, n(%)	 41(5)<br>15(94)	 39(5)<br>15(94)	
ATRA difference between limbs. Degrees, mean(SD)	7 (6)	7 (6)	
Muscle endurance n=7<br>-Work. Joules<br>-Reps. N(sd)	-	Injured<br>752(359)<br>75(25)	Uninjured<br>1130(321)<br>90(3)
One leg standing heel-rise ability. Yes(%)	-	10 (62.5%)	
CALM difference between limbs. cm(SD)	1.95(1.04)	1.94 (1.08)	
Cross sectional area difference. cm^2(SD)	1.15(0.21)	1.78 (0.79)	
Time for starting exercises<br>-Isometric (goal: day 7-14). Days(SD)(range)<br>-Seated heel-rise (goal: day 14-21). Days(SD)(range)<br>-Elastic band (goal: day 28-35). Days(SD)(range)	 9(2) (6-14)<br>17(6) (13-31)<br>30(4) (27-41)	-	

Data are presented as numbers (percentage), mean(SD) or median[interquartile range). ATRS Achilles Tendon total Rupture Score. IPAQ International Physical Activity Questionnaire. MET metabolic equivalent of task. TSK Tampa Scale of Kinesiophobia. ATRA Achilles tendon resting angle (difference between injured and healthy side). CALM Copenhagen Achilles Length measurement.
